# Statement of Retraction: Impacts of lipopolysaccharide on fetal lung developmental maturity and surfactant protein B and surfactant protein C protein expression in gestational diabetes mellitus rats

**DOI:** 10.1080/21655979.2024.2299600

**Published:** 2024-02-20

We, the Editors and Publisher of the journal *Bioengineered*, have retracted the following article:

Yue Yue Gao, Ziwei Zhang, Yan Wang, Dayong Zhou, Jinghua Zhang, Xiaoyu Chen, Xin Li & Qingliang Shao (2022) Impacts of lipopolysaccharide on fetal lung developmental maturity and surfactant protein B and surfactant protein C protein expression in gestational diabetes mellitus rats, Bioengineered, 13:1, 834-843, DOI: 10.1080/21655979.2021.2013099

Since publication, significant concerns have been raised about the integrity of the data and reported results in the article. When approached for an explanation, the authors did not provide their original data or any necessary supporting information. As verifying the validity of published work is core to the integrity of the scholarly record, we are therefore retracting the article. All authors listed in this publication have been informed.

We have been informed in our decision-making by our editorial policies and the COPE guidelines.

The retracted article will remain online to maintain the scholarly record, but it will be digitally watermarked on each page as ‘Retracted’.

